# Modulation of Ras signaling alters the toxicity of hydroquinone, a benzene metabolite and component of cigarette smoke

**DOI:** 10.1186/1471-2407-14-6

**Published:** 2014-01-05

**Authors:** Matthew North, Joe Shuga, Michele Fromowitz, Alexandre Loguinov, Kevin Shannon, Luoping Zhang, Martyn T Smith, Chris D Vulpe

**Affiliations:** 1 Department of Nutritional Science and Toxicology, University of California, Berkeley, California 94720, USA; 2 Division of Environmental Health Sciences, School of Public Health, University of California, Berkeley, California 94720, USA; 3 Department of Pediatrics and the Helen Diller Family Comprehensive Cancer Center, University of California, San Francisco, California 94115, USA

**Keywords:** Hydroquinone, *in vitro* micronucleus assay, *IRA2*, *NF1*, Ras, Yeast

## Abstract

**Background:**

Benzene is an established human leukemogen, with a ubiquitous environmental presence leading to significant population exposure. In a genome-wide functional screen in the yeast *Saccharomyces cerevisiae,* inactivation of *IRA2*, a yeast ortholog of the human tumor suppressor gene *NF1* (Neurofibromin), enhanced sensitivity to hydroquinone, an important benzene metabolite. Increased Ras signaling is implicated as a causal factor in the increased pre-disposition to leukemia of individuals with mutations in *NF1*.

**Methods:**

Growth inhibition of yeast by hydroquinone was assessed in mutant strains exhibiting varying levels of Ras activity. Subsequently, effects of hydroquinone on both genotoxicity (measured by micronucleus formation) and proliferation of WT and *Nf1* null murine hematopoietic precursors were assessed.

**Results:**

Here we show that the Ras status of both yeast and mammalian cells modulates hydroquinone toxicity, indicating potential synergy between Ras signaling and benzene toxicity. Specifically, enhanced Ras signaling increases both hydroquinone-mediated growth inhibition in yeast and genotoxicity in mammalian hematopoetic precursors as measured by an *in vitro* erythroid micronucleus assay. Hydroquinone also increases proliferation of CFU-GM progenitor cells in mice with *Nf1* null bone marrow relative to WT, the same cell type associated with benzene-associated leukemia.

**Conclusions:**

Together our findings show that hydroquinone toxicity is modulated by Ras signaling. Individuals with abnormal Ras signaling could be more vulnerable to developing myeloid diseases after exposure to benzene. We note that hydroquinone is used cosmetically as a skin-bleaching agent, including by individuals with cafe-au-lait spots (which may be present in individuals with neurofibromatosis who have a mutation in *NF1*), which could be unadvisable given our findings.

## Background

Benzene is ubiquitous in the environment due to its wide use in industry for the production of plastics, resins, dyes and detergents, and its presence in gasoline. Hydroquinone (HQ) is a primary oxidative metabolite of benzene. Globally, there is extensive population and occupational exposure to HQ [1]. Both benzene and HQ are also found in cigarette smoke and HQ is the most abundant pro-oxidant compound in cigarette smoke tar [2], though HQ is also found in the plasma of nonsmoker individuals living a Western lifestyle [3]. In addition to industrial exposure, there is significant dietary exposure to HQ in the form of arbutin, a HQ glucose conjugate [3]. Also, in some individuals dermal exposure can be significant as HQ is used as a skin-bleaching agent, to treat conditions such as hypermelanosis, vitiligo, and cafe-au-lait spots.

Benzene causes leukemia in humans (reviewed in [1]) and HQ itself causes DNA damage *in vitro*. HQ is capable of binding to both DNA and protein, and can induce oxidative stress and inhibit apoptosis [4-6]. HQ is formed through a multi-step process involving the action of Cytochrome P450s on benzene in the liver and perhaps other organs. HQ can travel to the bone marrow and is oxidized through both autoxidation and by myeloperoxidase (MPO) to highly toxic quinones [7], thus contributing significantly to benzene toxicity.

Ras proteins transduce cellular signals required for the control of cell growth and differentiation [8], and activating Ras mutations are present in ~30% of cancers [9,10]. The ERK effector kinases of the Ras/MAPK pathway modify diverse substrates which ultimately mediate cellular pathways controlling cell growth [11]. In a genome-wide functional screen using the yeast *Saccharomyces cerevisiae*, we previously identified *IRA2* as required for cellular tolerance to HQ treatment [6]. Ira2p is a Ras GTPase activating protein (GAP) that stimulates conversion of Ras proteins from their active GTP-bound state to the inactive GDP-bound state. Deletion of *IRA2* results in an increase in active Ras (and thus Ras signaling). Heterozygous germline mutations in *NF1,* the human homolog of *IRA2,* cause neurofibromatosis type I (NF1), a common developmental disorder with an incidence of 1 in 3000 live births [12]. NF1 belongs to a group of disorders referred to as the RASopathies (reviewed in [12]), caused by germline mutations in components of the RAS/MAPK pathway that all give rise to an increased cancer risk [12]. Individuals with NF1 are predisposed to benign and malignant tumors, which typically arise in cells derived from the embryonic neural crest [13,14]. Children with NF1 have a 200–500 fold risk of developing juvenile myelomonocytic leukemia (JMML), an aggressive myeloproliferative disorder (MPD) [15]. Consistent with its biochemical activity as a negative regulator of Ras signaling, JMML and other NF1-associated neoplasms frequently show somatic inactivation or the normal *NF1* allele [13,16]. Patients with NF1 are also at increased risk of developing myeloid leukemia and other genotoxin-induced malignancies [17], and homozygous mutations in *NF1* were recently reported in a subset of adult acute myelogenous leukemias (AML) [14].

Homozygous inactivation of a conditional mutant allele of murine *Nf1* in the hematopoietic compartment results in a progressive MPD that models JMML [18]. Heterozygous Nf1 mutant mice are also susceptible to myelodysplastic syndrome (MDS) and other genotoxin-induced cancers, and appear to be particularly sensitive to the mutagenic effects of radiation [19,20]. As with *IRA2* in yeast*, NF1* mutations lead to hyperactive Ras signaling [21,22], and are consistent with the high prevalence of somatic *NRAS* and *KRAS* mutations in these cancers. The association between hyperactive Ras signaling and leukemogenesis is particularly compelling in JMML and chronic myelomonocytic leukemia (reviewed in [9,23]).

Altered regulation of Ras signaling and exposure to benzene and its metabolites are both leukemogenic, but the sensitivity of *ira2*Δ to HQ represents, to our knowledge, the first observation of a potential synergism. Other examples of synergism between exogenous compounds and altered Ras signaling include the observation that lung carcinomas induced by cumene (a compound structurally similar to HQ) have greater malignant potential in mice with *K-ras* mutations [24], and also that benzene-induced mammary tumors in mice show an increased frequency of *H-ras* mutations [25]. However, an association between Ras signaling status and HQ toxicity has not been previously defined. Here we report our study demonstrating the interaction of mutations in both *IRA2* in yeast and *Nf1* in murine hematopoietic cells with the cellular consequences of HQ exposure.

## Results

### Modulation of Ras signaling in yeast alters the toxicity of hydroquinone

**Figure 1:**
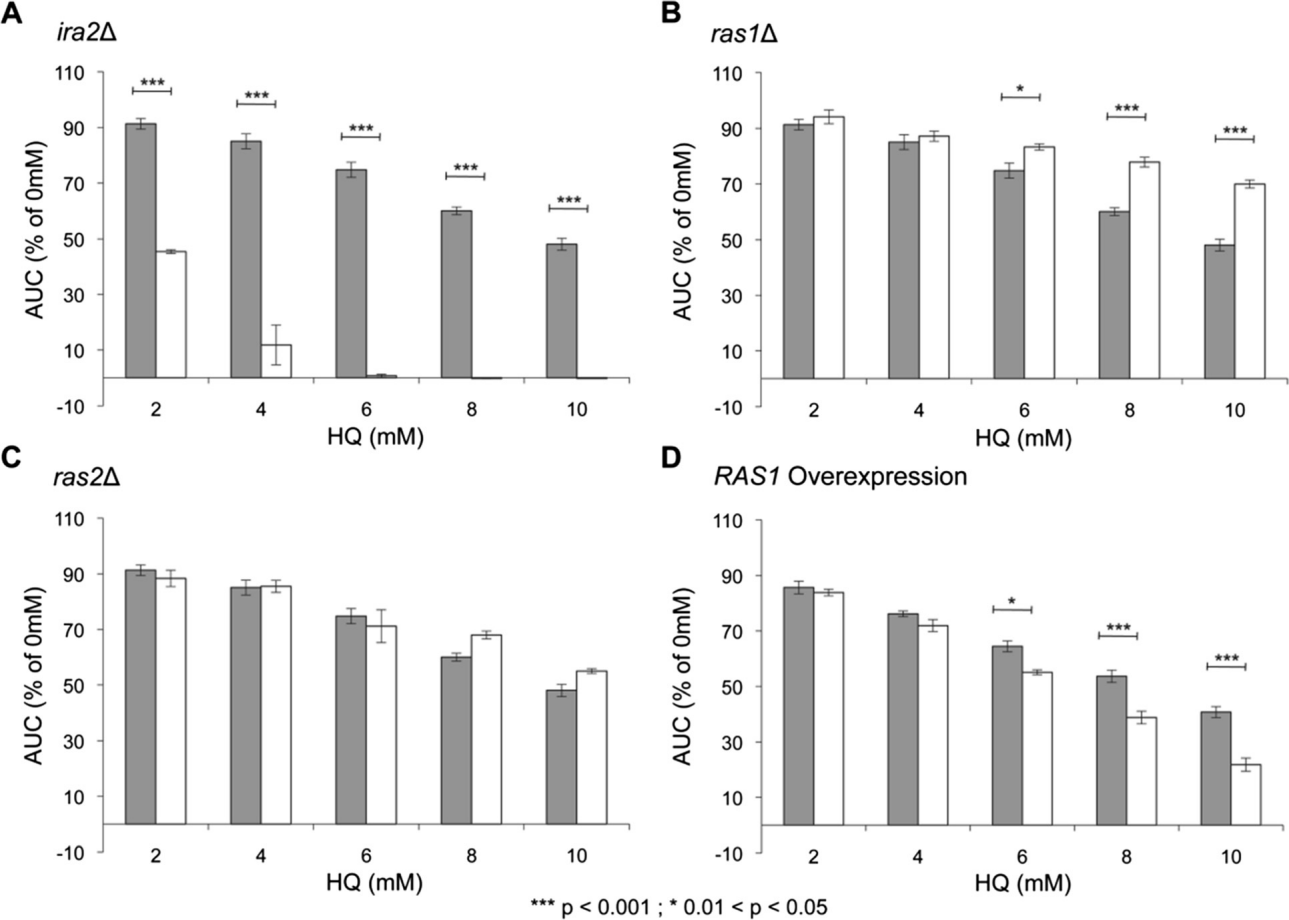
**The Ras status of yeast cells modulates the toxicity of HQ.** The Area Under Curve (AUC) was calculated for each strain after 24 h of exposure to the indicated doses of HQ. The bars represent mean AUC as a percentage of the untreated for each strain with standard error of three replicates. Sensitivity was determined by comparison to the wild type strain (gray bars = wild type; white bars = indicated deletion/overexpression strain). **A***ira2*Δ (increased Ras signaling) is sensitive to HQ. **B***ras1*Δ (reduced Ras signaling) is resistant to HQ. **C***ras2*Δ showed a response to HQ equivalent to WT. **D** Overexpression of *RAS1* (increased Ras signaling) results in sensitivity to HQ.

*IRA2* was previously identified in a genome-wide screen for genes required for HQ tolerance [6] and sensitivity to HQ was confirmed by analysis of the individual deletion strain (Figure 1A). *IRA2* is orthologous to the human tumor suppressor gene *NF1*, and expression of the human neurofibromin catalytic domain (GRD) in yeast recovers the phenotype of *ira2*Δ cells [26]. As Ira2p is a negative regulator of Ras (i.e. its deletion leads to increased Ras signaling due to decreased turnover of active-Ras) we tested other mutants in the Ras pathway that have altered signaling. Yeast has two Ras proteins, Ras1p and Ras2p, which are highly homologous to their mammalian counterparts. They exhibit some functional redundancy, like many *S. cerevisiae* paralogs, though Ras1p is specialized for proliferation-related signaling, whereas Ras2p regulates complex cellular differentiation (i.e. sporulation and filamentation) in response to nutrient starvation (reviewed in [27]). Deletion of *RAS1* results in increased tolerance to HQ treatment, whereas deletion of *RAS2* has no effect (Figure 1B, 1C and Additional file 1: Figure S1). To provide further mechanistic evidence for this observation that Ras signaling (through Ras1p) modulates HQ toxicity, we examined a *RAS1* overexpression strain, and this strain also exhibits increased sensitivity to HQ (Figure 1D).

### Increased genotoxicity is seen in murine bone marrow cells lacking Nf1 following HQ treatment

To determine if there was an influence of *Nf1* on DNA damage by HQ in mouse bone marrow cells, we assessed micronucleus (MN) formation using an *in vitro* erythroid MN assay [28]. Micronuclei are small fragments of nuclear membrane-encapsulated DNA formed by enucleation of cells with DNA damage during erythropoiesis. Measuring their presence *in vitro* can be used to sensitively detect compound genotoxicity [29] and requires fewer animals than other *in vivo* methods, while allowing for testing at an increased number of doses [28]. *Nf1* null mice die *in utero*, thus a *Cre/loxP* system was used to generate a conditional allele (*Nf1* flox) at the *Nf1* locus, resulting in ablation of neurofibromin in hematopoietic tissues following the induction of Cre expression using interferon gamma [18].

**Figure 2:**
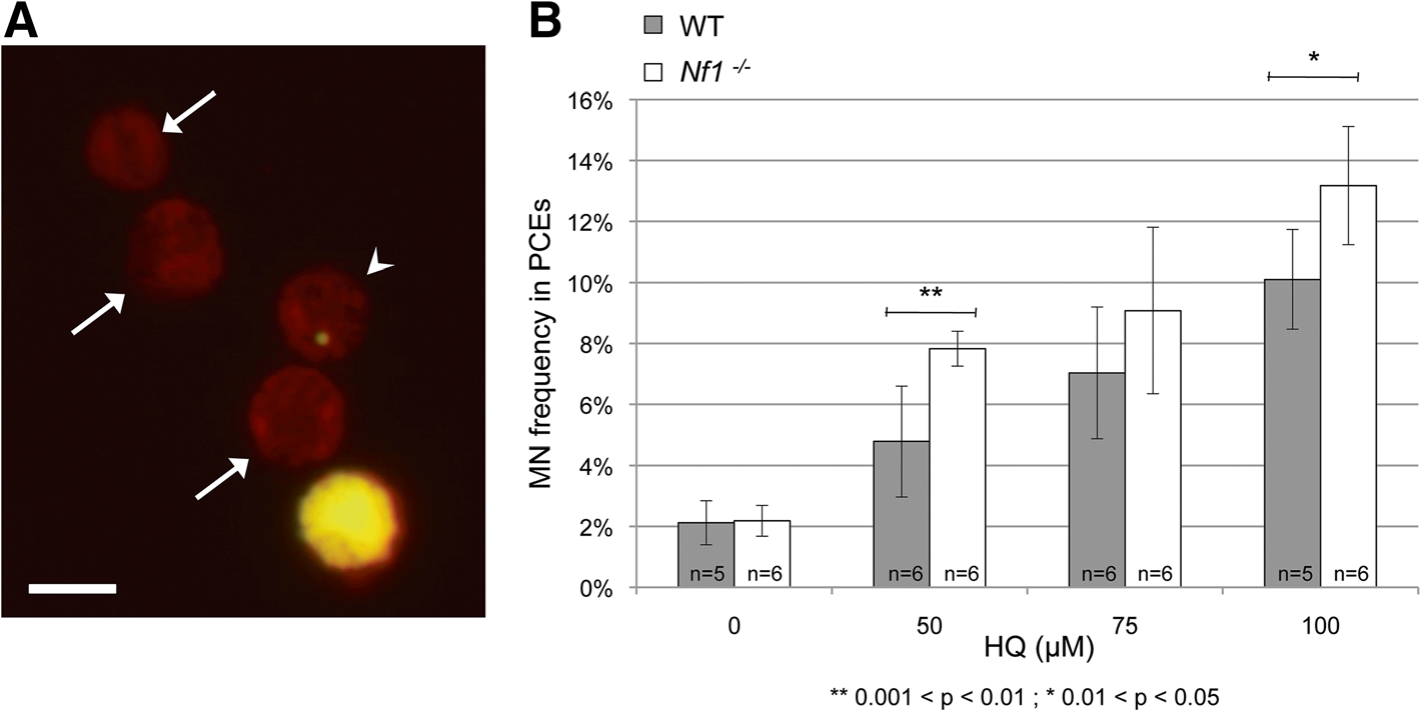
**Detection of genotoxicity through *in vitro *erythropoiesis. A** Representative micrograph of Day 2 harvested cultures at 100x magnification. The arrows indicate normally enucleated PCEs, and the arrowhead indicates a micronucleated PCE. Scale bar = 8 μM. **B***Nf1*^−/−^ Lin- BM cultures exhibit sensitivity to MN formation after *in vitro* exposure to HQ. The response of this erythropoietic culture system to HQ treatment is quantified by MN frequency in PCEs. Data presented is the mean of the “n” independent cultures ± SD.

We first established that HQ can induce MN *in vitro* as it does in the *in vivo* mammalian MN assay [29], and determined the appropriate dose range. Lineage-marker-negative (Lin^-^) BM was used as a starting population for *in vitro* erythropoietic MN experiments, and terminal erythropoiesis progressed normally as assayed by flow cytometry and histological examination (data not shown). Cultures were treated with HQ for 1 hour, starting 23 hours after seeding, and progressed normally through erythropoiesis. MN-PCEs appeared in both treated cultures and controls, but with greater frequency in treated cultures (Figure 2A).

Lin^-^ BM from *Nf1* mutant mice and their WT littermates was then used to test whether the *Nf1*^−/−^ genotype affected genotoxic sensitivity to HQ. There was a higher recovery of Lin^-^ BM cells from the *Nf1*^−/−^ mice (0.7%) than the WT littermates (0.3%). The greater recovery of Lin^-^ BM cells in the *Nf1*^−/−^ mice is likely indicative of chronic polyclonal hyperproliferation due to hypersensitivity to GM-CSF, which is mediated by increased and prolonged Ras activation [30]. Regardless, upon culturing, erythropoietic differentiation in *Nf1*^−/−^ cultures was indistinguishable from that of WT littermates. The MN frequencies observed *in vitro* for both the negative controls and the treated cultures were higher than the levels typically observed *in vivo*[19,20]. This increase may be caused by the relatively high rate of erythropoietic growth and lack of spleen function *in vitro*; increasing erythropoietic rates *in vivo* through bleeding [31] or exogenous erythropoietin (EPO) expression increases erythroid MN frequencies and has been shown to sensitize the *in vivo* assay to genotoxic exposure [32]. Cell viability at harvest was >85% for all cultures, but viable cell yields were lower for treated vs. control (data not shown). Cell proliferation and death were not explicitly measured, thus reductions in viable cell yields cannot be specifically attributed to cytotoxic or cytostatic mechanisms.

Both *Nf1*^−/−^ and WT cultures treated with hydroquinone at 50 μM, 75 μM, and 100 μM showed a significant dose-dependent increase (two-tailed unequal variance t-test, p < 0.05) in MN formation compared to the respective controls (Figure 2B – significance not indicated). A two-way ANOVA analysis found that there was an overall significant difference between the MN frequency found in the *Nf1*^−/−^ cultures and the corresponding WT cultures (p < 0.001). A two-tailed unequal variance Student’s t-test was employed to examine the differences at each dose. There was no significant difference in MN formation between the untreated cultures (p = 0.87) but there was a significantly greater induction of MN in the *Nf1*^−/−^ cultures than the WT cultures at both 50 μM (p < 0.01) and 100 μM (p < 0.05) (Figure 2B). This observation indicates that increased Ras signaling increases the genotoxicity of HQ.

### Murine bone marrow progenitor cells lacking Nf1 show increased proliferation relative to WT following treatment with HQ

**Figure 3:**
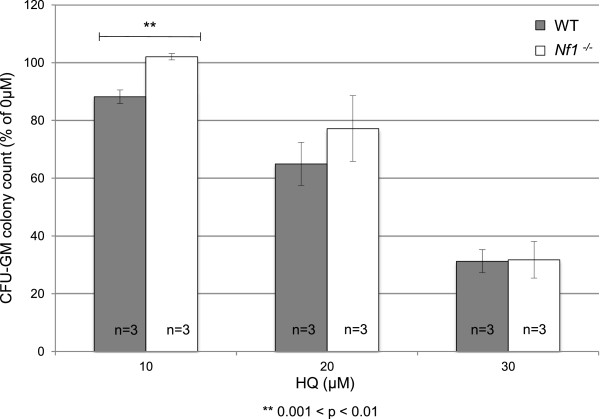
***Nf1***^**−/− **^**hematopoietic progenitors of the granulocyte and macrophage lineages are resistant to HQ-induced toxicity.** CFU-GM colony counts, relative to untreated controls, are plotted vs. HQ exposure level for cultures derived from both WT and *Nf1*^−/−^ BM. Bone marrow cells from each genotype were treated with HQ in MethoCult® capable of inducing growth of CFU-GM colonies and then cultured for 12 days. In both genotypes colony formation was reduced upon exposure to as little as 10 μM HQ. CFU-GM numbers continued to fall with increasing HQ exposure up to 30 μM HQ treatment, and at all doses the *Nf1*^−/−^ BM appeared resistant to HQ-induced toxicity when compared with BM from WT littermates.

To further investigate the possible role of *Nf1* in development of hematological malignancies such as leukemia following exposure to HQ, we measured the proliferative ability of myeloid hematopoietic progenitor cells after treatment. We assayed CFU-GM colony growth at several HQ dosage levels for WT and *Nf1*^−/−^ bone marrow cells and normalized this data to the untreated controls (Figure 3). HQ caused a dose-dependent decrease in the proliferative ability of progenitor cells in both WT and *Nf1*^−/−^ cells, in agreement with a previous study [33]. In both genotypes, colony formation was reduced upon exposure to as little as 10 μM HQ, and CFU-GM numbers continued to fall with increasing HQ exposure up to 30 μM HQ treatment (Figure 3). At higher doses of HQ (e.g. 100 μM), all colony formation was prevented by excessive toxicity to both genotypes (data not shown). Unexpectedly, *Nf1*^−/−^ progenitors demonstrated significantly increased survival to HQ exposure in comparison to WT (Figure 3) at 10 μM [p-value 0.0010]. We cannot ascribe these apparent differences to MPD in the *Nf1*^−/−^ mice because the inhibitory effects of HQ were normalized to growth in the absence of HQ for both genotypes. Moreover, the *Nf1*^−/−^ mice were euthanized at a relatively young age (12 weeks) and showed no overt evidence of myeloid disease as assessed by complete blood counts conducted on the harvest BM (data not shown). Thus, the difference observed is likely driven specifically by the HQ treatment.

## Discussion

Hydroquinone (HQ) is a benzene metabolite, component of cigarette smoke, and skin-bleaching agent. A genome-wide functional screen in the yeast *Saccharomyces cerevisiae* identified *IRA2* as required for tolerance to treatment with HQ.

*IRA2* is a yeast ortholog of the human tumor suppressor gene *NF1*, mutations in which cause the disease neurofibromatosis type I (NF1). Affected individuals are pre-disposed to developing cancers, including leukemia, due to increased Ras signaling [13]. The Ras protein family is involved in signal transduction, and their activation leads to growth, differentiation and cellular survival. *NF1* has been recently identified as important in the development of acute myelogenous leukemia (AML) [14], the same form of leukemia that is associated with exposure to benzene, an established human leukemogen. However, the association between Ras status and HQ toxicity has not been defined. Our previous identification of *IRA2* as required for HQ tolerance was the first observation of a potential synergism between Ras signaling and the toxicity of benzene. To gain further insight into this association we investigated the requirement of both *IRA2* in yeast and *Nf1* in murine hematopoietic cells for the response to HQ.

The sensitivity of yeast cells lacking *IRA2* to HQ was confirmed by analysis of the *ira2*Δ deletion strain. We found that while *ira2*Δ cells with increased levels of active Ras were more sensitive to HQ treatment, *ras1*Δ cells with decreased active Ras were less sensitive, and actually showed resistance to HQ. Consistent with these data, we also found that cells overexpressing *RAS1* (effectively phenocopying *ira2*Δ) show increased sensitivity to HQ. This confirmed that *ira2*Δ HQ sensitivity is not due to a specific property of Ira2p and provides substantial support for our hypothesis that the level of active Ras signaling modulates the toxicity of HQ.

How could increased Ras signaling cause an increase in HQ toxicity? The observation that modulation of HQ toxicity is specific to signaling through Ras1p (*ras2*Δ showed a response to HQ equivalent to WT) provides a potential explanation. Ras1p regulates progression of the cell cycle, and an increase in cell cycle rate could explain increased HQ toxicity. HQ has multiple cellular targets in yeast, including the cytoskeleton and vesicular transport, and it generates oxidative stress, causes lipid peroxidation and damages DNA [6]. The deleterious effects of these processes could be amplified in the context of increased cell cycle rate, and so would result in an increase in growth inhibition.

In order to study the significance of our findings in human health, we studied the relationship between *Nf1* and HQ tolerance in a murine model. We determined that the *Nf1*^−/−^ genotype renders mammalian bone marrow progenitor cells more sensitive to genotoxic damage by HQ, as measured by an increase in micronuclei formation. We have shown that HQ treatment causes a dose-dependent decrease in the number of CFU-GM colonies derived from both WT and *Nf1*^−/−^ progenitors. However, in contrast to the yeast studies, HQ treated *Nf1*^*−/−*^ cell showed increased proliferation in comparison to wild type cells. Our work and previous studies showed that untreated *Nf1*^*−/−*^ cells show increased proliferation due to hyperactive Ras [34], but even after normalization for this effect, HQ treatment at the doses used further increased proliferation of WT progenitors. We therefore propose that null cells may more rapidly divide down the differentiation lineages than WT (regardless of damage), and this higher proliferation of damaged cells could explain the greater MN frequency. By both increasing genetic damage and enhancing survival in *Nf1* mutant hematopoietic cells, HQ could facilitate the development of myeloid malignancies such as MDS and AML.

One possible explanation for this increased relative growth is that HQ could stimulate GM progenitor growth via ERK1/ERK2 and synergize with GM-CSF. Support for this idea comes from the observation that HQ stimulates GM-CSF-dependent proliferation of erythroleukemia cells and human CD34+ bone marrow cells via activation of ERK [35]. As noted above, in primary *Nf1*^*−/−*^ progenitor cells there is constitutive activation of Ras [34]. GM-CSF [34] mediates an increased and prolonged Ras activation [30] and *Nf1*^*−/−*^ cells, unable to modulate this effect, are hypersensitive to GM-CSF. Further, HQ acts synergistically with GM-CSF to induce proliferation of progenitor cells that would not normally be recruited by the cytokine [36]. Deletion of *Nf1* potentiates this effect due to increased Ras activation. Interestingly, 1,4-BQ (from HQ) also induces activation of ERK1 and ERK2 [37] in rat liver epithelial cells so it is possible that HQ could have a similar effect in bone marrow cells. Similarly, the hyperactive Ras effect of *Nf1*^*−/−*^ on erythroid progenitors acts specifically through the ERK pathway and not through other pathways regulated by Ras [38]. We therefore suggest that the combination of hyper-sensitization to GM-CSF in *Nf1*^*−/−*^ cells and activation of ERK by HQ could explain the increased proliferation of null cells relative to WT following HQ treatment. Alternatively or in addition, DNA damage caused by HQ may stimulate proliferation of GM progenitors in *Nf1*^*−/−*^ cells via p53 dependent mechanisms. Previous work found that the genotoxin cytarabine causes increased myeloid differentiation in bone marrow cells with a Ras mutation via a p53-dependent mechanism [39].

## Conclusions

Together our findings show that HQ toxicity is modulated by Ras signaling and that increased Ras signaling (by deleting *Nf1*) results in both an increased level of DNA damage detected in erythroid progenitors and increased survival of GM progenitors. HQ exposure and pre-existing increased Ras signaling could increase the likelihood of leukemic transformation. Importantly, our observations with HQ are highly consistent with observations in human patients and in mice showing that heterozygous *Nf1* inactivation cooperates strongly with genotoxins (particularly radiation) to induce myeloid malignancies and other secondary cancers [17,19]. In addition to NF1, individuals with Noonan syndrome and other RASopathies could be more vulnerable to developing myeloid diseases after exposure to benzene. Of particular concern is the use of hydroquinone as a skin-lightening agent by NF1 patients. HQ is given to NF1 patients to treat the characteristic cafe-au-lait pigmentation spots, which are a key diagnostic criterion of NF1. Our work indicates that the interaction between Ras signaling, sensitivity to toxicants, and the development of neoplasia needs to be given further consideration. Such studies may aid in the development of more protective standards for sensitive individuals to occupational and public exposures to carcinogens such as benzene.

## Methods

### Yeast strains and culture

Diploid yeast deletion strains used for growth analyses were of the BY4743 background (*MATa*/*MAT*α, *his3∆1*/*his3∆1*, *leu2∆0*/*leu2∆0*, *lys∆0*/*LYS2*, *MET15*/*met15∆0*, *ura3∆0*/*ura3∆0*, Invitrogen Corporation, Carlsbad, CA). The haploid yeast MORF (Movable ORF - [40]) strain for *RAS1* overexpression was of the Y258 background (*MATa*, *pep*4-3, *his*4-580, *ura*3-53, *leu*2-3,112 - Thermo Fisher Scientific Open Biosystems, Huntsville, AL). Growth was conducted in liquid rich media (1% yeast extract, 2% peptone, 2% dextrose, YPD) for deletion strain growth curve assays, and liquid synthetic complete media lacking uracil (SC-ura) using either 2% dextrose or 2% raffinose as a carbon source for pre-growths of the overexpression strain for growth curve assays (detailed below). Liquid rich media for induction of protein overexpression for growth curve assays contained both galactose and raffinose (1% yeast extract, 2% peptone, 2% galactose, 2% raffinose, YPGal + Raf).

### Yeast hydroquinone exposures

Hydroquinone (HQ) (Sigma-Aldrich, St Louis, MO) stock solutions were prepared fresh in sterile nuclease-free water (ISC BioExpress, Kaysville, UT) and protected from light.

### Yeast deletion strain growth curve assays

Yeast strains were pre-grown to mid-log phase, diluted to an optical density at 600 nm (OD_600_) of 0.0165, and dispensed into individual wells of a 48-well plate (non-treated polystyrene, Grenier Bio-One, Monroe, NC). HQ stock solution was added to the desired final concentrations with at least two replicates per dose. Plates were incubated in a GENios microplate reader (Tecan, Durham, NC) set to 30°C with intermittent shaking. OD_595_ measurements were taken at 15-minute intervals for a period of 24 hours. Raw absorbance data were averaged for all replicates, background corrected, and plotted as a function of time. The area under the curve (AUC), used as a measure of growth, was calculated with Excel 2008 (Microsoft Corporation, Redmond, WA) and expressed as a percentage of the control. AUCs were compared with two-way ANOVA followed by Bonferroni post-tests, using GraphPad Prism version 5.01 (GraphPad Software, La Jolla, CA). Data for each strain is derived from three independent biological replicates.

### Yeast RAS1 overexpression strain growth curve assays

The yeast *RAS1* overexpression strain was pre-grown overnight to stationary phase in SC-ura 2% dextrose, diluted 1:100 in SC-ura 2% raffinose and grown overnight again to alleviate glucose repression. Cells were then diluted in YPGal + Raf to induce protein overexpression, and grown for 5 hours to mid-log phase. Cells were subsequently diluted to an optical density at 600 nm (OD_600_) of 0.0165 in YPGal + Raf, and dispensed into wells of a 48-well plate. Hydroquinone treatment, plate measurement and data processing were all carried out in the same manner as for the deletion strain growth curve assays.

### Mice

*Mx1-Cre, Nf1*^*flox/flox*^ mice (129SV × C57BL/6 J) were generated and somatic *Nf1* inactivation was induced as described elsewhere [18]. We refer to hematopoietic cells from these animals as *Nf1*^*−/−*^ throughout this paper. Cells from wild type (WT) littermates were used as controls in all experiments. Mice were maintained in the sterile animal care facility at the University of California, San Francisco (UCSF), and were fed pelleted chow and acidified water ad libitum. All experimental procedures involving mice were approved by the UCSF Committee on Animal Research.

### Murine cells

Tissue was harvested from mice at ~12 weeks. Bone marrow (BM) cells were isolated from the hind legs and were mechanically dissociated by pipetting in Iscove’s modified Dulbecco’s medium (IMDM) with L-glutamine/4% FBS (IMDM – Lonza, Walkersville, MD). Single-cell suspensions were prepared by passing dissociated cells through 70 μm cell strainers. BM cells were counted using a hemocytometer.

### Erythropoietic culture

Total BM cells were labeled with biotin-conjugated α-Lin Abs, consisting of α -CD3e, α -CD11b, α -CD45R/B220, α -Ly6G/Ly6C, and α -TER-119 Abs (2 μl of each Ab/10^6^ cells; BD Pharmingen, San Diego, CA), and Lineage-marker-negative (Lin^-^) cells were purified through a 0.3-in StemSep negative selection column as per the manufacturer’s instructions (StemCell Technologies, Vancouver, BC, Canada). Purified cells were seeded in fibronectin-coated (2 μg/cm^2^) tissue culture treated 24-well polystyrene plates (BD Falcon, BD Biosciences San Jose, CA) at a cell density of 10^5^ cells/ml. On the first day, purified cells were cultured in IMDM containing basal supplements consisting of: 15% FBS, 1% detoxified BSA, 200 μg/ml holotransferrin (Sigma, St Louis, MO), 10 μg/ml recombinant human insulin (Sigma), 10^-4^ M β-mercaptoethanol, 50 units/ml penicillin G, and 50 μg/ml streptomycin; as well as soluble erythropoietic factors including erythropoietin (Epo - Amgen, Thousand Oaks, CA) at 7.5 units/ml and stem cell factor (SCF - R & D Systems, Minneapolis, MN) at 10 ng/ml. For all cultures, media was replaced with erythroid-differentiation medium (EDM) (IMDM with 20% FBS, and 10^-4^ M β-mercaptoethanol) after 1 day of culture. At harvest, suspended cells were removed from culture wells by pipetting, and the culture well was then incubated in PBS/10% FBS/5 mM EDTA for 5 minutes at 37°C to dissociate adherent cells. Dissociated cells were then removed from the culture well by pipetting and combined with the suspended cell fraction from the same culture well for analysis. Viable cell counts, based on trypan blue exclusion, were conducted using a hemocytometer.

### Genotoxic treatment of erythropoietic cultures

Lin^-^ BM was cultured in 500 μl of medium per culture well according to the method described for erythropoietic culture. Cultures were treated one hour before being washed and fed with EDM, as earlier described. HQ solutions were prepared immediately before treatment and protected from light to minimize degradation and decreased reactivity. HQ (Sigma) was first dissolved in warm PBS to make a 10 mM stock solution. This 10 mM solution was diluted in 4°C PBS to produce 7.575 mM and 5.05 mM solutions, and 5 μl of a solution was added to each culture to expose them to the targeted concentrations of HQ (100 μM, 75 μM, and 50 μM).

### Cytospin preparation and cytological staining

Approximately 2×10^4^ cells per culture were centrifuged onto slides for 2 minutes at 800 rpm (Statspin Cytofuge 2; Iris Sample Processing Westwood, MA) and air-dried. For acridine orange staining, cells were fixed in 25°C methanol for 10 minutes and stained in acridine orange (Sigma) at a concentration of 20 μg/ml in staining buffer (19 mM NaH_2_PO_4_ and 81 mM Na_2_HPO_4_) for 10 minutes at 4°C. Following acridine orange staining, slides were protected from light, washed for 10 minutes in 4°C staining buffer, air-dried, and stored at 4°C until microscopic examination and scoring was complete.

### Histological imaging and quantification

Slides were examined by using an Axioplan 2 microscope (Carl Zeiss MicroImaging GmbH, Germany) and representative micrographs were acquired by using Axiocam MRm (Carl Zeiss). Micrographs of acridine orange stained cells were acquired by using a 63x oil-immersion objective and fluorescence (100 W Hg lamp excitation). Cytological slides were examined blind, and differential cell counting was used to enumerate relevant cell types and thus quantify the frequency of micronucleated polychromatic erythrocytes (MN-PCEs) among total PCEs (>2,000 PCEs scored per slide).

### Statistics

To determine the statistical significance of mean comparisons, distributions were first checked for normality by using the Shapiro–Wilk's test with STATA 11 (StataCorp, College Station, TX). Normal data sets were then subjected to two-way ANOVA analysis. Interaction between dose and breed was examined but found to be insignificant and so was not used in the model. Two-tailed Student’s *t* tests were executed by using the data analysis tool in Excel (Microsoft Corporation, Redmond, WA). Unequal variance was assumed to increase confidence in significant p-values.

### Progenitor assays

Colony forming unit granulocyte/macrophage (CFU-GM) progenitors were grown in MethoCult® (StemCell Technologies, Cat #03434) according to manufacturer’s instructions (Technical Manual Cat# 28405). Briefly, BM cells were suspended at 4×10^5^/ml in IMDM/2% FBS and then 200 μl of this suspension was added to 4 ml aliquots of MethoCult® M3434, which were then vortexed briefly. A fresh solution of 10 mM HQ (Sigma) was then prepared in warm PBS and sterile filtered through a 0.2 μm membrane (Pall Life Sciences, Ann Arbor, MI). This 10 mM HQ stock solution was then further diluted to give 600 μM, 400 μM, and 200 μM solutions, and then 200ul of one of these solutions was used to treat each 4 ml aliquot of MethoCult® at a concentration of 30 μM, 20 μM, or 10 μM. After delivery of HQ, the MethoCult® was again vortexed briefly and the tubes were left to stand for 5 minutes to allow bubbles to dissipate. A 16G blunt-end needle and 3 ml syringe were then used to draw up the treated MethoCult® and dispense 1.1 ml into each of three 35 mm petri dishes (StemCell Technologies, Cat# 27100). These 35 mm culture dishes (3 per genotype/treatment replicate) were then tilted and rotated gently to evenly distribute MethoCult® and placed in a 100 mm culture dish that also contained an uncovered 35 mm culture dish containing 3 ml of sterile water to maintain humidity. The cultures were placed in an incubator and maintained at 37°C, 5% CO_2_ in air, and >95% humidity for 12 days, then colonies were scored. The scoring scientist was blinded to the identity of each culture (genotype/treatment). Two independent preparations of each culture condition (combination of genotype/treatment) were used in each trial, and the entire experiment was repeated three further times using independent sets of *Nf1*^−/−^/WT littermates.

## Competing interests

MTS has received consulting and expert testimony fees from law firms representing both plaintiffs and defendants in cases involving claims related to exposure to benzene.

## Authors’ contributions

MN, JS, MF, KS, LZ, MTS and CDV conceived and designed the experiments. MN, JS, and MF performed the experiments. MN, JS, MF and AL analyzed the data. KS, LZ, MTS and CDV contributed reagents and materials. MN, JS, MF, KS, LZ, MTS and CDV wrote the paper. All authors read and approved the final manuscript.

## Pre-publication history

The pre-publication history for this paper can be accessed here:

http://www.biomedcentral.com/1471-2407/14/6/prepub

## Supplementary Material

Additional file 1: Figure S1 *ras2*Δ is significantly more sensitive to HQ than *ras1*Δ. The Area Under Curve (AUC) was calculated for each strain after 24 h of exposure to the indicated doses of HQ. The bars represent mean AUC as a percentage of the untreated for each strain with standard error of three replicates. Sensitivity was determined by comparison to the wild type strain (gray bars = *ras1*Δ; white bars = *ras2*Δ). Click here for file
