# Supplementary material for: Modulation of Ras signaling alters the toxicity of hydroquinone, a benzene metabolite and component of cigarette smoke
Source: BMC Cancer. 2014 Jan 5;14:6. doi: 10.1186/1471-2407-14-6 (PMC3898384; doi:10.1186/1471-2407-14-6)
Supplement: Additional file 1: Figure S1 — ras2Δ is significantly more sensitive to HQ than ras1Δ. The Area Under Curve (AUC) was calculated for each strain after 24 h of exposure to the indicated doses of HQ. The bars represent mean AUC as a percentage of the untreated for each strain with standard error of three replicates. Sensitivity was determined by comparison to the wild type strain (gray bars = ras1Δ; white bars = ras2Δ). [file 1471-2407-14-6-S1.pdf]

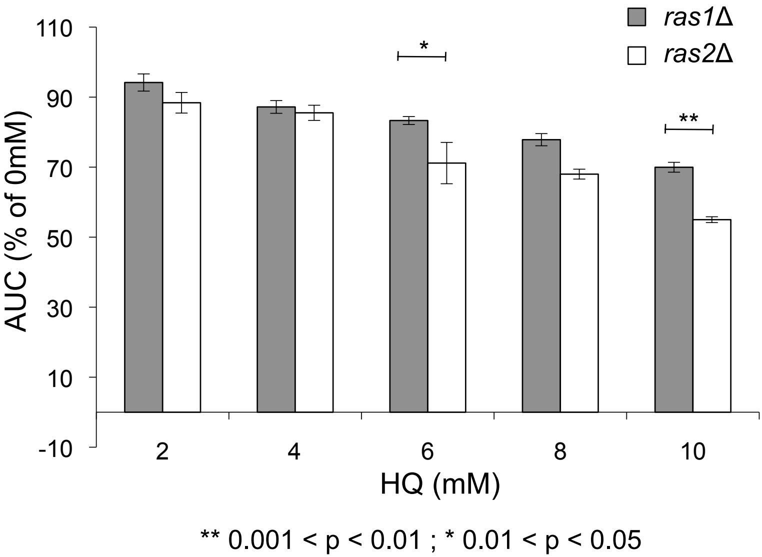


**Supplemental Figure 1. *ras2*Δ is significantly more sensitive to HQ than *ras1*Δ.** The Area Under Curve (AUC) was calculated for each strain after 24h of exposure to the indicated doses of HQ. The bars represent mean AUC as a percentage of the untreated for each strain with standard error of three replicates. Sensitivity was determined by comparison to the wild type strain (gray bars = *ras1*Δ; white bars = *ras2*Δ).
